# A single nucleotide polymorphism in EZH2 predicts overall survival rate in patients with cholangiocarcinoma

**DOI:** 10.3892/ol.2013.1559

**Published:** 2013-09-02

**Authors:** ELISA PAOLICCHI, PAOLA PACETTI, ELISA GIOVANNETTI, ANDREA MAMBRINI, MASSIMO ORLANDI, FRANCESCO CREA, ANTONELLO A. ROMANI, ROBERTA TARTARINI, ROMANO DANESI, GODEFRIDUS J. PETERS, MAURIZIO CANTORE

**Affiliations:** 1 Department of Internal Medicine, Division of Pharmacology, University of Pisa, Pisa I-56126, Italy; 2 Department of Oncology, Carrara Civic Hospital, Piazza Sacco e Vanzetti, Carrara I-54033, Italy; 3 Department of Medical Oncology, VU University Medical Center, Amsterdam, Amsterdam 1081HV, The Netherlands; 4 Department of Surgical Sciences, Division of Surgical Clinic and Organ Transplant, University of Parma, Parma I-43100, Italy

**Keywords:** polycomb, EZH2, cholangiocarcinoma, ECX regimen, polymorphism

## Abstract

Cholangiocarcinoma (CCA) is a deadly disease arising from the malignant transformation of cholangiocytes. Enhancer of zeste homolog 2 (EZH2) is overexpressed in poorly differentiated CCA. Functional single nucleotide polymorphisms (SNPs) in this gene may affect the role of EZH2 in cholangiocarcinogenesis and chemoresistance. The aim of the current study was to evaluate the correlation between EZH2 SNPs and clinical outcome. Using PROMO3.0, GeneCard and MicroSNiper, 4 EZH2 SNPs with functional relevance in CCA were selected *in silico*. These SNPs were studied in genomic DNA extracted from the blood samples of 75 patients with advanced CCA, who were treated with epirubicin-cisplatin-xeloda (ECX regimen). SNP genotyping was performed with specific PCR assays. The rs887569 TT genotype was correlated with a significantly longer overall survival (OS; TT vs. CT-CC, P=0.026). Moreover, the TT genotype revealed a trend toward a significant association with a reduced risk of mortality (HR, 0.59; 95% CI, 0.33–1.05; P=0.075), by multivariate analysis. These results support future studies on the role of rs887569 EZH2 SNP as a possible predictive marker of OS in advanced CCA patients.

## Introduction

Cholangiocarcinoma (CCA) is a malignancy arising from the biliary tract epithelium (1). CCA is rare, with an annual incidence of 2–3 cases per 100,000 individuals in the Western population (2). However, it is the second most common primary hepatic malignancy, with recent epidemiological studies suggesting a progressively increasing incidence in Western countries (3–5). Anatomically, CCA is classified as intrahepatic (IH-CCA) or extrahepatic (EH-CCA). IH-CCA arises from the intrahepatic ducts, which extend from the periphery of the liver to the second-order bile ducts within the liver (6). EH-CCA is observed and defined in three different growth patterns: periductal infiltrating, papillary or intraductal and mass forming (7). The high mortality rate of CCA is mainly due to its aggressive behavior. Indeed, the majority of these tumors are diagnosed at a late stage of disease progression, precluding surgical therapies (8,9). Furthermore, CCA is characterized by a marked resistance to chemotherapy (10,11). Several drugs have been tested in phase II studies in unresectable CCA (5-fluorouracil, gemcitabine methasulfon-m-anisidide, cisplatin, rifampicin, mitomycin C and paclitaxel) with partial response rates below 9% and average survival shorter than 12 months (8). Therefore, studies for the identification of key factors that play a critical role in tumor chemosensitivity/resistance for the selection of patients with the highest likelihood of responding to these therapies are urgently required.

Polycomb group (PcG) proteins are epigenetic chromatin modifiers involved in cancer development and their roles are now being evaluated in numerous human malignancies (12). PcG proteins are essential for cancer stem cell (CSC) self-renewal. PcG members are organized in two main protein complexes: Polycomb repressive complex 1 and 2 (PRC1, PRC2) (13). PRC2 is required in the initial stage of silencing, through the histone H3 Lys 27 trimethylation (14) which contributes to the recruitment of PRC1. PRC1 is required for stable maintenance of the initiated PcG gene silencing, through histone H2A ubiquitylation (14), on specific target loci. Enhancer of zeste homolog 2 (EZH2) is the catalytic subunit of PRC2; while B-cell-specific Moloney murine leukemia virus integration site 1 (BMI-1) contributes to the ubiquitin E3 ligase activity in PRC1. EZH2 is overexpressed in poorly differentiated CCA (15). Several genetic and epigenetic factors may be involved in the deregulation and modulation of key signaling pathways in tumor aggressiveness and chemoresistance. The abnormal expression of EZH2 is involved in the tumorigenic processes and is regarded as a potential marker of aggressive types of cancer with poor prognoses (16,17). Previous studies have demonstrated that EZH2 contributes to the epigenetic silencing of several target genes that control cell growth and proliferation, including E-cadherin, Rb and p16 (18). The overexpression of EZH2 may induce hypermethylation of the promoter of the p16 gene, reducing the expression of p16, which is a key step in the multistep cholangiocarcinogenesis from hepatolithiasis to intraepithelial neoplasia (19).

A recent study described 26 single nucleotide polymorphisms (SNPs) in the EZH2 locus, including SNPs correlated with lung cancer risk (20). Genetic analysis of germinal variants may be an easy-to-perform prognostic tool, readily transferable into the clinic. Therefore, the study of candidate polymorphisms of EZH2 as biomarkers of clinical outcome may provide effective prognostic markers in CCA patients. The aim of the current study was to evaluate a correlation between EZH2 SNPs and clinical outcome in CCA patients.

## Materials and methods

### Patients

A total of 75 patients with histologically confirmed unresectable biliary tract cancer were enrolled in this retrospective pharmacogenetic single-center study at the Department of Oncology of Carrara Civic Hospital (Carrara, Italy), between February 2004 and November 2010. CCA patients were treated upfront with intravenous or intra-arterial cisplatin (Platinol^®^, Bristol-Myers Squibb, Roma, Italy) and epirubicin (Pharmorubicin^®^, Pfizer Italia S.R.L., Latina, Italy) and oral capecitabine (Xeloda^®^, Roche S.p.A, Milano, Italy; ECX regimen). This study was approved by the ethics review board of Carrara Hospital. Informed consent was obtained from all the patients.

### DNA isolation

Genomic DNA was extracted from peripheral venous blood samples (5 ml) from an antecubital vein of 75 CCA patients and stored anonymously at −20°C in the Laboratory of VU University Medical Center, Department of Medical Oncology (Amsterdam, The Netherlands). Genomic DNA was isolated through the QIAamp DNA mini kit (Qiagen, Venlo, The Netherlands). The purity and quantity of DNA obtained were measured by spectrophotometer NanoDrop^®^-1000-Detector (NanoDrop-Technologies, Wilmington, DE, USA). The absorbance was read at 260 and 280 nm and the contamination by proteins was estimated through the calculation of 260/280 ratio.

### In silico analysis

A total of 26 previously described EZH2 SNPs were functionally tested by the appropriate software. SNPs were screened through *in silico* characterization based on functional relevance [missense mutation, transcription factor binding (TFB), miRNA binding]. In particular, the PROMO3.0 (http://alggen.lsi.upc.es/cgibin/promo_v3/promo/promoinit.cgi?dirDB=TF_8.3) in which human factors and binding sites were considered, with a maximum matrix dissimilarity rate of 15 (21,22), GeneCard (http://www.genecards.org/) and MicroSNiper (http://cbdb.nimh.nih.gov/microsniper) software were used (23).

### SNP genotyping

EZH2 SNPs g.148525904C>G (rs2302427), g.148519011C>T (rs6464926), g.148517456T>G (rs17171119) and g.148505302 C>T (rs887569) were analyzed with real-time PCR. Applied Biosystems SNP genotyping assays were used for reactions. The PCR assays were performed using 20 ng of genomic DNA diluted in 5.94 μl DNase RNase free water, 6.25 μl of TaqMan Universal Master Mix (Applied Biosystems, Foster City, CA, USA) with AmpliTaq Gold and 0.3125 μl of the assay mix (specific primers and probe) in 12.5 μl total volume. The allelic content of each sample in the plate was determined by reading the generated fluorescence.

### Statistical analysis

All SNPs were examined for Hardy-Weinberg equilibrium (24). Overall survival (OS) and time to progression (TTP) curves were obtained through the Kaplan-Meier method and the log rank test was used to compare the survival distributions. P<0.05 was considered to indicate a statistically significant result. The Cox regression model was used to test the effect of g.148505302 C>T SNP and prognostic factors on OS.

## Results

### Patient characteristics and responses

Patient characteristics are summarized in Table I. The median ECOG performance status and median Ca19.9 level at diagnosis were 1 and 204 IU/ml, respectively. Of the 74 evaluable patients, 3 complete responses (CR) were observed (4.1%), while a partial response (PR) was observed in 10 of 74 evaluable patients (13.5%), stable disease (SD) in 34 patients (45.9%) and progressive disease (PD) in 27 patients (36.5%). A median follow-up of 42.3 months revealed that the median OS was 14.9 months (8.2–21.6) and the 1-year survival rate was 56%.

### In silico characterization of g.148505302 C>T, g.148525904C>G, g.148519011C>T and g.148517456T>G

**Figure 1:**
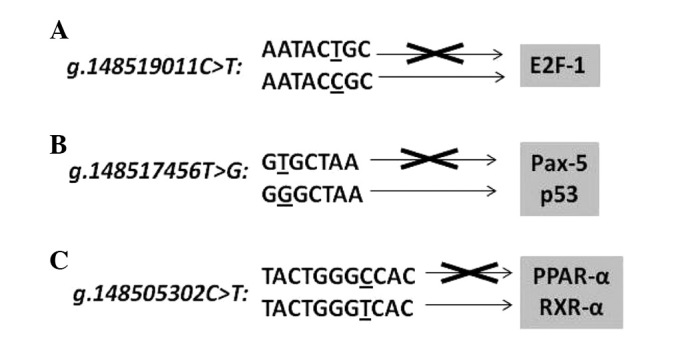
Identification of putative TFBS in DNA sequences. EZH2 SNPs may be responsible for differential TFB. (A) g.148519011 CC allows E2F-1 binding; (B) g.148517456 GG allows Pax-5 and tumor suppressor p53 binding; and (C) g.148505302 TT allows PPAR-α/RXR-α complex binding. TFBS, transcription factor binding sites; EZH2, Enhancer of zeste homolog 2; SNPs, single nucleotide polymorphisms; TFB, transcription factor binding.

Among the 26 SNPs in the EZH2 locus described by Yoon *et al*(20), only 1 SNP (g.148525904C>G) is located on an exon (exon 6) and is responsible for an amino acid change (histidine/aspartate). Conversely, 25 SNPs are located in EZH2 non-coding regions and they may affect EZH2 expression by affecting miRNA, transcription regulator binding or mRNA splicing. However, no SNPs affecting miRNA binding or splicing were identified, while the PROMO 3.0 software detected noteworthy correlations between the allelic variants of 3 of these EZH2 SNPs and TFB sites (Fig. 1). In g.148505302 C>T, the T allele creates a binding site for the peroxisome proliferator-activated receptor (PPAR)-α/retinoid-X-receptor (RXR)-α. The C allele of g.148519011C>T creates a binding site for E2F-1 and the G allele of g.148517456T>G creates a binding site for Pax-5 and p53. All these TFs are expressed in CCA cell lines (25–28).

### SNP genotyping

The 4 SNPs selected (Table II) were successfully evaluated in all available DNA samples. The g.148505302 C>T, g.148519011C>T and g.148525904C>G SNPs were in Hardy-Weinberg equilibrium. Conversely, the g.148517456T>G SNP did not follow the Hardy-Weinberg equilibrium. However, all SNPs had frequencies comparable to those observed in Caucasian populations reported in Pubmed Reference-SNP (RefSNP).

### EZH2 SNP correlation with clinical outcome

**Figure 2:**
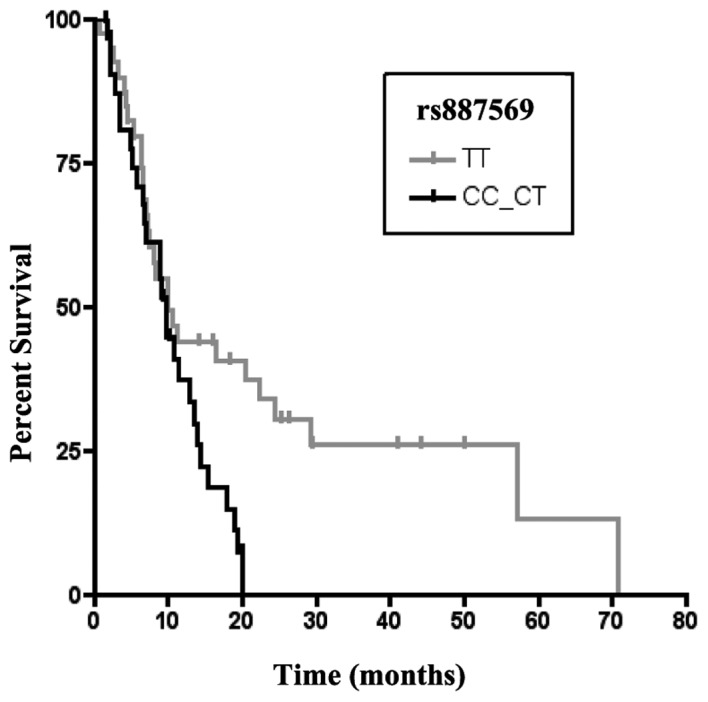
OS according to g.148505302 C>T genotype (rs887569). OS, overall survival.

No significant correlations were identified between the g.148519011C>T, g.148525904C>G and g.148517456T>G SNPs and clinical outcome, while the g.148505302 C>T (rs887569) SNP had a significant association with OS. In particular, the patients harboring the TT genotype had a significantly longer OS (TT vs. CT-CC, P=0.036; Fig. 2). Moreover, the TT genotype revealed a trend-like correlation with OS (P=0.075) in multivariate analysis.

## Discussion

The present study revealed that the g.148505302 T allele is associated with a longer OS in CCA patients. The g.148505302 C>T SNP is located in intron 19 of the EZH2 gene (20). Through *in silico* analysis it was revealed that the T allele creates a binding site for the PPAR-α/RXR-α heterodimer. The PPARs were identified in 1990 by Issemann and Green (29). PPARs are ligand-activated transcription factors that directly influence the transcription of target genes (30). Three related PPAR isotypes have been identified (PPAR-α, -β/δ and -γ) (31). PPAR-α binds to DNA as a heterodimeric complex with RXR-α (32). This complex binds to a specific sequence in regulatory regions of target genes, known as peroxisome proliferator response element (PPRE), with two copies of a hexameric nucleotide sequence, TGACCT-like (32). Several studies have suggested differential mechanism underlying the role of PPAR-α in cancer, including key roles in modulation of cell-cycle genes, cell proliferation and cellular apoptosis (33). In accordance with these hypotheses, the g.148505302 T allele, which binds the PPAR-α/RXR-α complex, may trigger mechanisms involved in apoptosis and cell proliferation inhibition by downregulation of the EZH2 oncogene. EZH2 is a transcriptional repressor involved in cell proliferation (19). Overexpression of EZH2 is associated with aggressive and metastatic disease in various types of cancer (34), including CCA. In particular, EZH2 is not expressed in the cholangiocytes or hepatocytes of livers without tumors, but is overexpressed in poorly differentiated carcinoma (35). Therefore, EZH2 expression may be a predictor of the biological aggressiveness and poor prognosis in CCA (36). Cell culture studies have confirmed the expression of EZH2 mRNA in CCA cells, but not in normal cells (35). These studies have also demonstrated that when EZH2 is decreased by suberoylanilide hydroxamic acid (SAHA; a histone deacetylase inhibitor) treatment, the tumor suppressors p16, E-cadherin and p21 are activated (35). Previous studies have shown that EZH2 expression had a stepwise increase in aggressive and invasive CCA (36,37). Since EZH2 drives CSC self-renewal and is associated with poor prognosis in most malignancies, it is conceivable that these cells contribute to the maintenance of the tumoral mass and are implicated in CCA chemoresistance.

EZH2 expression and activity may be affected by functional polymorphisms. SNP genotyping is particularly attractive for tumors detected in the advanced stages, including CCA, since it is an easy-to-perform analysis. This analysis may be performed with a small volume of biological fluids (e.g. 200 μl of blood specimens) in less than 24 h.

The current study demonstrates how a candidate EZH2 SNP may be a novel biomarker correlated with clinical outcome in CCA patients. Due to the relatively small sample size and retrospective design, further studies are required in order to validate the prognostic role of this SNP in CCA. Our *in silico* prediction should also be extended by appropriate molecular analyses, which go beyond the scope of the present analysis. In addition, the possibility that the g.148505302 C>T SNP is in linkage disequilibrium with other polymorphic variants, which may be responsible for the prognostic significance of this marker, cannot be ruled out.

In conclusion, to the best of our knowledge, this is the first study to show an EZH2 SNP having a significant impact on CCA outcome, possibly through its role in the PPAR-α/RXR-α complex interaction with EZH2. If these results are confirmed by larger prospective studies, this EZH2 polymorphism may be useful for predicting the clinical outcome in CCA patients.

## Figures and Tables

**Table I:** Patient characteristics.

Characteristics	No. of patients
General	
Total	75
Evaluable disease	74
Median age (range), years	62.3 (26–80)
Gender	
Male	44
Female	31
ECOG	
0	35
1	30
2	10
Tumor diagnosis	
Intrahepatic cholangiocarcinoma	56
Gallbaldder carcinoma	11
Common bile duct	8
Sites of metastases	
Liver	60
Involvement <50%	40
Involvement >50%	20
Lymph nodes	10
Peritoneum	7
Local disease recurrence	6
Other	4
Median Ca19.9 level (range, IU/ml)	204 (0–11400)

Involvement represents the percentage of liver tissue with metastatic cells.

**Table II:** Position and functional characteristics of the investigated SNPs.

SNP	Position	Change	Comments
g.148525904C>G	Exon 6	C:Histidine/G:Aspartate	
g.148519011C>T	Intron 8	C: E2F-1 TFB	TF expressed in CCA (23)
g.148517456T>G	Intron 8	G: Pax-5 and p53 TFB	TF expressed in CCA (24)
g.148505302 C>T	Intron 19	A:PPAR-α/RXR-α TFB	TF expressed in CCA (25)

SNP, single nucleotide polymorphism; TFB, transcription factor binding; PPAR-α, peroxisome proliferator-activated receptor-α; RXR-α, retinoid-X-receptor-α; TF, transcription factor; CCA, cholangiocarcinoma.
